# Impact on Metabolism Generated by Surgical and Pharmacological Interventions for Weight Loss in Women with Infertility

**DOI:** 10.3390/metabo15040260

**Published:** 2025-04-10

**Authors:** Paulo César Gete Palacios, Alberto Moscona-Nissan, Renata Saucedo, Aldo Ferreira-Hermosillo

**Affiliations:** Unidad de Investigación Médica en Enfermedades Endocrinas, Instituto Mexicano del Seguro Social, Centro Médico Nacional Siglo XXI, Hospital de Especialidades, México City 06720, Mexico; pcgete@hotmail.com (P.C.G.P.); albertomoscona@gmail.com (A.M.-N.); renata.saucedo@imss.gob.mx (R.S.)

**Keywords:** obesity, female fertility, pregnancy, female sexual function

## Abstract

Obesity increases the risk of anovulation, insulin resistance, hyperandrogenism, and endometrial dysfunction, resulting in women with infertility and increasing preconceptional and pregnancy complications. Bariatric surgery has been described as the most effective intervention for obesity, with improved fertility outcomes. However, its invasive nature increases the potential of nutritional deficiencies and the need for a delayed conception post-surgery. On the other hand, pharmacological treatments such as glucagon-like-peptide 1 receptor agonists offer non-invasive alternatives with promising results in body weight, improving insulin sensitivity and restoring ovarian function. However, their use must be discontinued before conception due to potential fetal risks. Other available pharmacological treatment options encompass topiramate, phentermine, and Orlistat. The choice of treatment must be individualized considering cost-effectiveness, accessibility, obesity severity, reproductive goals, and associated risks within each patient. A multidisciplinary approach is essential to optimize metabolic and reproductive health in obesity and infertility. This review will examine the impact on metabolism when comparing surgical and pharmacological interventions for weight loss in women with infertility.

## 1. Introduction

Obesity represents a global public health issue associated with a broad spectrum of complications that encompass cardiovascular disease, neoplasms, gastrointestinal disorders, and reproductive health consequences [1]. The Organization for Economic Co-operation and Development (OECD) estimates yearly the prevalence of overweight and obesity in the population aged over 15 years in different countries. In 2020, the United States reported a prevalence of 67.5% of overweight and obesity, Canada of 54.4%, and Spain of 50.2%, while Mexico had a measured prevalence of 74.1% [2]. Some factors that contribute to obesity worldwide include unhealthy diets, lack of physical activity, alcohol consumption, and tobacco use. Furthermore, modern lifestyles characterized by increased urbanization, sedentarism, and processed food consumption have raised the prevalence of overweight and obesity in the recent decades [3].

Obesity is more prevalent among women, imposing significant clinical and metabolic alterations on fertility, reducing their ability to conceive both naturally and through assisted methods. Obesity is associated with a higher risk of spontaneous abortions, congenital anomalies, premature births, fetal death, and perinatal complications such as gestational diabetes and hypertension. Moreover, it raises the probability of requiring surgical delivery and the risk of post-surgical complications, including wound infections and thromboembolism [4].

A complex and multifactorial relationship exists between obesity and women’s infertility, with several mechanisms that lead to negative reproductive outcomes. These encompass hypothalamic–pituitary–ovarian (HPO) axis dysfunction, hyperinsulinism and peripheral insulin resistance (IR), hyperandrogenism, chronic inflammation, adipocyte dysfunction, and altered ovarian and endometrial function. Obesity is associated with overproduction of factors such as leptin, free fatty acids, and cytokines by excess adipose tissue, which can alter ovarian function, oocyte maturation, and receptivity of the endometrial epithelium. In some cases, IR generates a state of hyperandrogenism and functional hyperestrogenism, which results in anovulation and reduced endometrial receptivity, leading to infertility [5].

Therefore, preconceptional weight loss treatment can enhance reproductive outcomes due to improved fertility and menstrual and ovulatory function. Currently, bariatric surgery remains as the most effective treatment for obesity and its associated diseases, providing benefits in cardiovascular and renal health and decreasing mortality [6].

The aim of this article is to analyze the metabolic impact of surgical and pharmacological interventions for weight loss in women with infertility. A comprehensive search was conducted in PubMed for original, peer-reviewed articles addressing bariatric surgery and pharmacological interventions for obesity in women with infertility. We searched for articles in English using the following search terms: Obesity, Bariatric Surgery, Female Infertility, Female Sexual Function, Pregnancy Outcomes.

## 2. The Impact of Obesity on Female Infertility

The prevalence of obesity varies depending on race, country, and socioeconomic status, but is higher in women [7]. In 2012, an analysis by the US National Health and Nutrition Examination Survey (NHANES) reported an obesity prevalence of 31.8% in women of reproductive age from 20 to 39 years [8]. Overweight and obesity (BMI >25 kg/m^2^ and >30 kg/m^2^) in women are associated with irregular menstrual periods, difficulty conceiving, and an increased frequency of miscarriages. The OECD Social Indicators describe worldwide fertility trends across the decades. Based on their published data, fertility rates have declined over the years from 1980 to 2020. Most countries around the world have seen decreased fertility rates. Israel is the only country with persistent rates, remaining over 3.1 since 1980. Other countries with persistent rates above 2.1 are Indonesia, South Africa, and Saudi Arabia. Meanwhile, the countries with the lowest rates of fertility include Korea, China, and Japan [9].

Females with obesity require an increased time to conceive, being correlated with BMI [10,11]. In fact, the risk of non-conceiving after a year attempting increases by sevenfold in women with obesity class III (BMI ≥40 kg/m²) when compared with patients with a normal BMI [11]. Furthermore, despite the proven efficacy of assisted reproductive technology (ART) in women with a normal BMI, its efficacy decreases in women with obesity [8,9]. A reduction rate of 68% in live births has been documented in women with obesity undergoing ART when contrasted to those with a normal weight [11,12,13,14].

## 3. Underlying Mechanisms in Obesity and Infertility

A highly frequent factor beneath obesity-related infertility is polycystic ovary syndrome (PCOS). A high proportion of patients with infertility and obesity are diagnosed with this condition and exhibit clinical features of hyperandrogenism. Globally, distinct criteria are used for the diagnosis of PCOS, causing variations in the reported prevalence [15,16,17]. Nevertheless, the European Society for Human Reproduction and Embryology and the American Society for Reproductive Medicine (ESHRE/ASRM) Rotterdam consensus criteria are the most commonly used. The latter define the diagnosis of PCOS upon the “presence of two of three following criteria: Oligo and or an-ovulation, clinical and biochemical evidence of hyperandrogenism, and presence of polycystic ovarian morphology on ultrasound” [16,17]. Studies have shown that PCOS affects 8–13% of reproductive-aged women worldwide, while the prevalence rates of PCOS are 12–21% among women with obesity [18]. Also, PCOS imposes a heavy risk on the development of IR, type 2 diabetes, and metabolic syndrome. [19].

IR is present in a fraction of patients with PCOS, impairing glucose uptake while other intracellular insulin actions remain intact. In the ovaries, insulin acts on its own receptor and in the insulin-like growth factor-1 (IGF-1) receptor, enhancing the effects of luteinizing hormone (LH) and promoting ovarian steroidogenesis [17]. The estimated prevalence of overweight and obesity in women with PCOS ranges from 40 to 80% [17,20]. In addition to IR, hyperandrogenism is considered a contributor that leads to infertility. Sex hormone-binding globulin (SHBG) carries androgens at a high affinity. In the presence of obesity, lower SHBG levels cause hyperandrogenism, with insulin and IGF-1 involved in its decrease [1,17].

A myriad of hormones involved in energy metabolism also have reproductive functions. In addition to storing energy, adipose tissue secretes adipokines such as leptin, adiponectin, resistin, and ghrelin, which have several implications on fertility by causing chronic inflammation and altering estriol levels and oocyte quality. At the gastrointestinal tract, leptin inhibits the appetite and increases energy expenditure, and ghrelin induces an appetite [21,22]. Additionally, insulin and IGF play important roles in fertility [23]. IR is a key mechanism in the pathogenesis of PCOS, and thus insulin-sensitizing medications such as biguanides are frequently used to enhance fertility outcomes in affected women [17,23,24].

## 4. Molecular Aspects of Obesity

When delving into the molecular mechanisms through which obesity leads to negative fertility outcomes, the constant aromatization of androgens into estrogen has been observed, leading to HPO axis dysregulation [25]. Furthermore, development of IR due to obesity induces the dysregulation of glucose homeostasis with impairment of cellular processes, such as glucose uptake by GLUT4 and glycogen synthesis [26]. Those processes are caused by the activation of the protein kinase C theta, leading to abnormal levels of AKT2 [26,27,28]. On the other hand, serum free fatty acid levels are elevated due to lipolysis [29,30] in patients with obesity. These alterations increase the levels of insulin and inflammatory markers [27,28], interfering with normal HPO axis functioning through high LH levels and increased androgen synthesis.

Moreover, additional factors that impact female fertility encompass oxidative stress, circulating lipids, and blood glucose, which tend to be increased in patients with obesity [31,32,33,34]. Oxidative stress increases various pregnancy complications such as spontaneous abortion, premature rupture of the fetal membranes, and preeclampsia. However, more studies are required, as a certain amount of oxidative stress is required for normal fetal development [35,36]. Some studied effects of increased oxidative stress include altered oocyte quality and changes in the ovarian microenvironment. Additionally, circulating lipids as increased LDL also increases the infertility risk [37]. In fact, patients with dyslipidemia present a longer duration of infertility, reduced antral follicle count, and increased FSH levels when compared with patients without dyslipidemia [38].

## 5. Preconceptional Obesity and Pregnancy Complications

It has been established that the risk of miscarriage increases by 30% in women with obesity [11,39]. Some of the complications associated with obesity during pregnancy encompass gestational diabetes and preeclampsia [11]. On the other hand, the accumulation of adipose tissue represents a challenge to both physicians attempting to calculate fetal size and detect congenital abnormalities and mothers attempting to identify reduced fetal movements, resulting in delayed identification of complications [11,39,40,41]. A meta-analysis demonstrated an increased risk of neural tube defects, macrosomia, hydrocephalus, and cardiovascular, craniofacial, and musculoskeletal anomalies in children of women with obesity when compared to women with normal weight [11].

Maternal obesity increases the risk of gestational diabetes up to 4–9 times when compared with women with normal weight [8]. Other complications associated encompass hypertensive states during pregnancy such as preeclampsia, exacerbating the long-term risk of cardiovascular complications, a doubled risk of ischemic heart disease, and a four-fold increase in the risk of developing hypertension. Finally, cardiovascular implications may also be present in the newborn [11,42,43,44,45,46,47].

Although labor and delivery guidelines vary globally, maternal obesity carries complications and special peripartum and postpartum considerations [8]. Delivery through cesarean section, need to induce labor, and postpartum hemorrhage are more frequent in women with obesity [8,42,43,44,45,46,47].

## 6. Lifestyle Modifications to Improve Female Obesity

Lifestyle modifications represent a cornerstone for weight management, even in the case of women with infertility. The main modifications encompass the implementation of a balanced diet, physical activity, improved sleep habits, and reduction in stress [48]. Induction of a negative energy balance is critical for weight loss; it can be promoted by different strategies such as a calorie-restriction diet, intermittent fasting, and time-restricted feeding [49,50]. The intervention of a multidisciplinary team of health professionals including a nutritionist is fundamental for suggesting individualized diet plans in combination with physical activity, taking into consideration a series of aspects such as the current weight, age, gender, comorbidities, patient needs, and goals [51]. There is evidence that the combination of healthy lifestyle behaviors such as reduced alcohol consumption, smoking cessation, a healthy diet, and physical activity can reduce the overall risk of mortality by 66% [52]. To promote physical activity and prevent non-communicable diseases, the World Health Organization recommends 150 min of moderate-level exercise or 75 min of intense physical activity per week [52,53]. Current dietary recommendations include increased consumption of grains, legumes, fruits, vegetables, and fish while reducing the intake of red meat and processed foods [54].

Ruiz-González et al. conducted a systematic review and meta-analysis comparing the efficacy of exercise, diet, and pharmacological interventions in managing the BMI, ovulation, and hormonal profile in reproductive-aged women with overweight or obesity. Strategies integrating physical activity with diet and pharmacotherapy were successful in inducing ovulation and improving hormonal profiles, while dietary interventions alone or paired with weight loss drugs showed added benefits for reducing BMI. The most significant improvements were observed with comprehensive lifestyle modifications and pharmacological therapies [55].

## 7. Pharmacological Treatment in Obesity and Female Infertility

Given the high prevalence of obesity among females of reproductive age, the aforementioned impact on female fertility and pregnancy outcomes, and the invasive nature of bariatric surgery procedures, pharmacological approaches have gained much attention in the last years [56]. Currently, there are several known medications for the management of obesity and weight maintenance. Nevertheless, physicians should bear in mind lifestyle modifications, including critical recommendations for patients with obesity.

**Figure 1:**
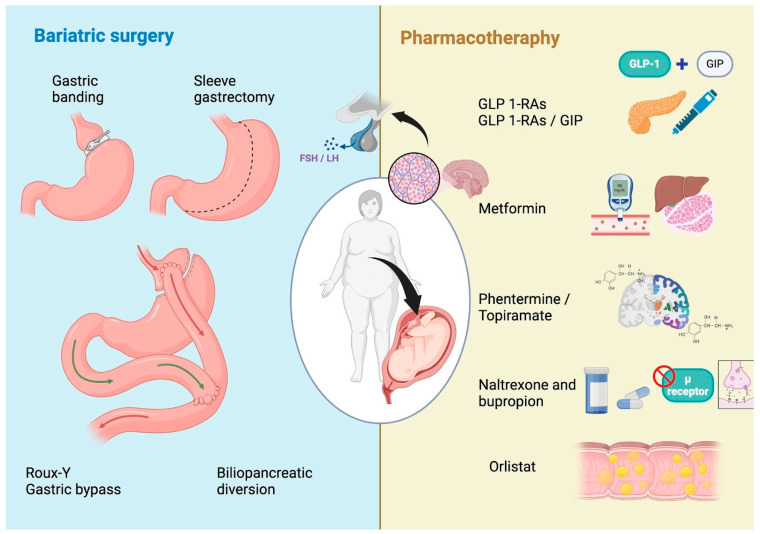
Surgical and pharmacological interventions for weight loss in women with infertility.

Despite the proven efficacy of bariatric surgery, pharmacotherapy aims to mimic the physiological effects of surgery with less invasive and modern interventions. Among the therapies for weight control, some of the most safe and modern interventions include Orlistat, phentermine/topiramate, naltrexone/bupropion, and GLP1-RAs or GLP1-RAs/GIP, as shown in Figure 1. Although no medication has been proven to be as effective as surgical treatment for obesity, encouraging results have been found [57]. However, it is extremely important to know each of the pharmacological options as well as their probable adverse effects in order to choose the best treatment for each patient, also considering her fertility desires.

The first group of pharmacological agents that can be used in women with obesity and infertility are insulin-sensitizing agents such as oral biguanides [17]. Metformin is known as the first-line treatment for type 2 diabetes and has proven to reduce mortality and prevent the development of diabetes [58]. Metformin has been widely studied and used as an insulin-lowering agent in the context of PCOS, characterized by a state of hyperinsulinemia, hyperandrogenism, and altered folliculogenesis [17]. In patients with PCOS, metformin increases the ovulation rate, improving pregnancy rates and live-birth rates, as demonstrated by Morin-Papunen et al. in a multicenter, double-blind, placebo-controlled trial [59]. There are also in vitro data pointing to the effect of metformin in decreasing ovarian androgen production [60,61]. However, Tang et al. studied the effect of metformin alone versus in combination with lifestyle modifications regarding changes in menstrual cycles, finding that metformin does not improve weight loss or menstrual frequency, while lifestyle modifications improve menstrual function after weight reduction [17,60].

Next, glucagon-like peptide receptor agonists (GLP1-RAs) are another group of medications currently approved by the FDA for treatment of diabetes and obesity, such as liraglutide (Saxenda, Victoza) and semaglutide (Rybelsus, Ozempic, Wegovy), which differ in dosage, administration, and effectiveness [56,62]. Currently, this group of medications represents the most effective pharmacotherapy for weight loss. GLP-1 is physiologically released after carbohydrate consumption in the L cells of the small intestine along with Peptide YY [34]. GLP-1 is the prototypical incretin hormone, acting by satiety induction and enhancing insulin secretion and glucose uptake [58]. Moreover, incretins increase the sensation of fullness at a central level and slow stomach emptying [56]. GLP1-RAs have been studied for their impact on obesity and diabetes outcomes, given their ability to reduce food intake and alleviate metabolic syndrome [58,62,63].

Additionally, GLP1-RAs have been demonstrated to improve kidney and cardiovascular outcomes and reduce mortality in patients with type 2 diabetes and chronic kidney disease [64,65]. In fact, GLP1-RAs have shown a significant reduction in major adverse cardiovascular events (MACEs) depending on their type and dosage [66].

GLP1-RAs are considered a category C drug in pregnancy for their teratogenicity in rat and rabbit controls, the main reason why their use in pregnant women should be avoided [67]. It is recommended to discontinue treatment with GLP-1RAs such as semaglutide and tirzepatide at least two months prior to conception [64,68]. Liraglutide should be discontinued 10–14 weeks prior to conception since it has a shorter half-life [69,70,71]. Clinical studies have demonstrated that GLP1-RAs increase the likelihood of pregnancy in women suffering from PCOS or obesity [69,70,71]. For example, Graham et al. conducted a study with the main purpose of determining the long-term effects of prenatal GLP1-RAs’ activation on the behavior of female mice prior to conception and through the prenatal period. They found that GLP1-RAs’ activation during pregnancy had no negative effects on maternal outcomes, highlighting the potential safety of GLP1-RAs during pregnancy [70]. Although GLP1-RAs have been studied as the main treatment for obesity, there are no prospective studies, cohort studies, or population-based studies in women with infertility [71,72].

Twincretin or dual therapy of GLP1-RAs with GIP (gastric inhibitory polypeptide) has been described as one of the most novel obesity and diabetes therapy [73]. Tirzepatide has demonstrated a weight loss of more than 15% in individuals with obesity [74,75]. Its outcomes related to sexual function in women have not been studied. As with GLP1-RAs, its use is contraindicated in pregnancy, as well as ongoing acute pancreatitis, type 1 diabetes, gastroparesis, inflammatory bowel disease, medullary thyroid cancer, multiple endocrine neoplasia (MEN), and hypersensitivity reactions [73]. The most frequent side effects of GLP1-RAs or twincretin therapy are related to gastrointestinal symptoms including nausea, diarrhea, and vomiting classified from mild to moderate during the dose-escalation period [76].

Contrave, which combines naltrexone and bupropion, is another FDA-approved therapy for weight loss. This drug combination includes an µ-opioid receptor antagonist in combination with a norepinephrine and dopamine receptor inhibitor [77]. Contrave has been studied for suppressing appetite and speeding up metabolism, increasing energy expenditure [56,77]. This medication has shown to be a more comprehensive and effective treatment in patients with obesity in the context of emotional or binge eating [56,78]. The main adverse effects of contrave encompass gastrointestinal effects such as nausea, constipation, and vomiting; central nervous system effects such as tremors, dizziness, and headaches; and psychiatric disorders such as insomnia, anxiety, hallucinations, and depression. Other symptoms such as fatigue, palpitations, and increased blood pressure have been described [79].

On the other hand, Qsymia, another FDA-approved therapy since 2012 for weight loss, combines phentermine with topiramate in an extended-release formulation. Phentermine is a sympathomimetic amine anorectic that acts at a central level and induces catecholamine release. Phentermine is considered to induce satiety at the hypothalamic level by releasing norepinephrine and increasing leptin [80,81]. Topiramate is an anticonvulsant and migraine prophylactic drug that stabilizes the neuronal membrane by targeting voltage-activated calcium and sodium channels, increasing GABA activity and regulating excitatory neurotransmitters such as glutamate [80,81,82]. Both medications reduce food cravings and suppress appetite, representing a good combination for weight loss when administered concomitantly [74]. Some side effects associated with phentermine and topiramate include a dry mouth, constipation, paresthesia, insomnia, and dysgeusia [80,81,82]. However, topiramate has been associated with fetal malformations including a cleft lip and cleft palate. Therefore, its high teratogenic effect makes it a less recommended therapy for obesity in women with infertility [81,82].

Finally, Orlistat represents another pharmacotherapeutic approach to treat obesity. Its mechanism is based on the inhibition of the pancreatic and gastric lipases, which are essential for fat absorption [76]. Thus, the main mechanism of Orlistat to treat obesity is by inhibiting fat absorption and reducing caloric uptake [56,82]. Expected side effects of this drug encompass steatorrhea, flatulence, and increased defecation, which can be ameliorated by increased fiber consumption. Currently, Orlistat is approved by the FDA for weight loss; however, its benefit on fertility outcomes remains limited [82].

To date, several trials have examined the effect of Orlistat in women with infertility, such as the study by Wang et al. and the FIT-PLEASE trial [56,83,84]. The first was a randomized, double-blinded, placebo-controlled trial in infertile women with overweight or obesity scheduled for in vitro fertilization, with Orlistat and placebo arms over 1–3 months. The trial results showed that Orlistat is effective at inducing weight loss, but no significant differences regarding live births, conception, or pregnancy loss were observed [56,83]. Regarding the FIT-PLEASE trial, lifestyle modifications alongside Orlistat proved to decrease weight by 7% on average. Nonetheless, live birth rates, pregnancy rates, and the time to conception remained unchanged when compared to an intense physical activity-based intervention without Orlistat [56,84].

## 8. Bariatric Surgery in Obesity and Female Infertility

Bariatric surgery is a group of procedures performed on the stomach or intestine in order to treat obesity. It can be classified as restrictive (gastric banding and sleeve gastrectomy), malabsorptive (biliopancreatic diversion), and mixed (Roux-Y gastric bypass), as shown in Figure 1. Current recommendations for bariatric surgery include patients with a BMI higher than or equal to 40 kg/m^2^ or with a BMI between 35 and 39.9 kg/m^2^ with additional comorbidities, in whom lifestyle changes and pharmacological interventions have produced insufficient effects [85,86,87].

Bariatric surgery leads to an improvement in menstrual cycles and fertility outcomes. Rapid weight loss can be achieved with bariatric surgery through decreasing the amount of consumed food and serum glucose levels, and through rearrangement of the gastrointestinal anatomy. A key mechanism of the clinical effectiveness of bariatric surgery is increasing the secretion of GLP-1 and Peptide YY [87]. It induces a rapid increase in SHBG levels, causing reduced concentrations of androgens and an increase in FSH after surgery, improving ovulation rates. Nevertheless, conception should be avoided between 12 and 24 months after surgery, due to nutritional and micronutrient deficiencies that could impact fetal health [88].

There is limited work comparing bariatric surgery procedures regarding fertility outcomes [88]. The principal mechanisms through which bariatric surgery improves fertility outcomes are by regulating the menstrual cycles, improving the secretion of sex hormones, increasing ovulatory capacity, and improving fertility in women with anovulation who have overweight or obesity [85,87,88]. Bastounis et al. evaluated 38 women with obesity of reproductive age, a year after undergoing vertical banded gastroplasty, finding a significant decrease in estradiol and total and free testosterone, while increases in FSH and SHBG levels were observed [87,89]. An increase in LH, FSH, and SHBG, as well as a decrease in testosterone and dehydroepiandrosterone sulphate (DHEAS), were found after biliopancreatic diversion with a duodenal switch [87,90]. Furthermore, regarding metabolic syndrome features, higher rates of remission of diabetes, hypertension, and dyslipidemia have been registered after bariatric surgery [91].

## 9. Medical Therapy vs. Bariatric Surgery

Given the critical roles of PCOS and obesity as key underlying mechanisms in female infertility, Samarasinghe et al. conducted the first randomized controlled trial to compare the safety and efficacy of vertical sleeve gastrectomy versus medical therapy regarding ovulation rates in patients with PCOS, obesity, and oligomenorrhoea or amenorrhea [92]. The findings include an increased median number of spontaneous ovulations in the surgical group when compared to the medical group, with ovulatory events increased by 2.5 in the medical treatment group during follow-up after a year. Moreover, bariatric surgery led to better anthropometric, cardiometabolic, quality of life, and psychological outcomes [92]. However, more complications were reported in the surgical group, with no long-term sequelae occurring [92].

Another study comparing bariatric surgery versus medical therapy regarding cost-effectiveness was conducted by Haseeb et al. They contrasted pharmacological treatment for weight loss in patients with obesity class II with semaglutide versus bariatric surgery with endoscopic sleeve gastroplasty (ESG). ESG consists of inserting a suturing device through the patient’s throat in order to suture the stomach, which is less invasive than traditional sleeve gastrectomy [56,93]. They concluded that ESG was more cost-effective than semaglutide. Price threshold analyses revealed lower costs of ESG, theoretically requiring the cost of semaglutide to decrease by threefold to eliminate its dominance [93]. In Table 1, we compare the markers and outcomes of fertility among patients treated with pharmacological and surgical approaches. In Table 2, we summarize the effectiveness of bariatric surgery and pharmacological treatment in women with obesity and infertility.

**Table 1:** Effect of interventions on markers and fertility improvement.

Marker/Outcome	Bariatric Surgery	Pharmacological Treatment
Weight Loss	Significant and sustained weight reduction [85,86,87,88,89,90,91].	Moderate weight reduction, varies by drug [56,57,58,59,60,61,62,63,64,65,66,67,68,69,70,71,72,73,74,75,76,77,78,79,80,81,82,83,84].
Menstrual Regularity	Improves menstrual cycles significantly [88].	Improves menstrual cycles, especially with insulin-sensitizing drugs [56,57,58,59,60,61,62,63,64,65,66,67,68,69,70,71,72,73,74,75,76,77,78,79,80,81,82,83,84].
Ovulatory Function	Strong improvement in ovulation rates [88].	Moderate improvement, varies by medication [56,57,58,59,60,61,62,63,64,65,66,67,68,69,70,71,72,73,74,75,76,77,78,79,80,81,82,83,84].
Testosterone Levels	Significant reduction post-surgery [88].	Moderate reduction with insulin-sensitizing and anti-androgen drugs [56,57,58,59,60,61,62,63,64,65,66,67,68,69,70,71,72,73,74,75,76,77,78,79,80,81,82,83,84].
FSH and LH Levels	Increase in FSH and LH, improving ovarian function [87,88,89,90].	Variable effects, dependent on medication type [56,57,58,59,60,61,62,63,64,65,66,67,68,69,70,71,72,73,74,75,76,77,78,79,80,81,82,83,84].
SHBG Levels	Significant increase, reducing free androgens [87,88,89,90].	Moderate increase with some treatments [56,57,58,59,60,61,62,63,64,65,66,67,68,69,70,71,72,73,74,75,76,77,78,79,80,81,82,83,84].
Insulin Sensitivity	Major improvement due to weight loss and metabolic changes.	Improves with insulin-sensitizing drugs [56,57,58,59,60,61,62,63,64,65,66,67,68,69,70,71,72,73,74,75,76,77,78,79,80,81,82,83,84].
GLP-1 and Peptide YY	Significant increase, enhancing satiety and metabolism [87,88].	Increases with GLP-1 receptor agonists [56,57,58,59,60,61,62,63,64,65,66,67,68,69,70,71,72,73,74,75,76].
Risk of Pregnancy Complications	Reduced with weight loss but needs monitoring for nutritional deficiencies [88].	Drugs must be discontinued before conception due to risks [56,57,58,59,60,61,62,63,64,65,66,67,68,69,70,71,72,73,74,75,76,77,78,79,80,81,82,83,84].

**Table 2:** Effectiveness of bariatric surgery vs. pharmacological treatment on women with obesity and infertility.

Aspect	Bariatric Surgery	Pharmacological Treatment
Indication	Patients aged 18–60 with BMI >40 kg/m² or BMI 35–39.9 kg/m² with comorbidities [85,86,87].	Women with obesity and infertility seeking weight loss and improved ovulation [56].
Mechanism of Action	Reduced intake, changes in intestinal anatomy, and increased GLP-1 and PYY [87].	Appetite suppression, fat absorption reduction, and improved insulin sensitivity [56,57,58,59,60,61,62,63,64,65,66,67,68,69,70,71,72,73,74,75,76,77,78,79,80,81,82,83,84].
Impact on Fertility	Improved menstrual cycle regulation, increased ovulation, and enhanced fertility [87,88].	Reduction in hyperinsulinemia, improved ovulation, and fertility restoration with certain medications [56,57,58,59,60,61,62,63,64,65,66,67,68,69,70,71,72,73,74,75,76,77,78,79,80,81,82,83,84].
Effect on Sex Hormones	Decrease in estradiol, testosterone, and DHEA-S; increase in FSH, LH, and SHBG [87,88,89].	Reduction in ovarian androgens, potential reversal of polycystic ovary morphology with GLP-1 RA [56,57,58,59,60,61,62,63,64,65,66,67,68,69,70,71,72,73,74,75,76,77,78,79,80,81,82,83,84].
Examples of Treatments	Gastric banding, sleeve gastrectomy, Roux-Y gastric bypass, and biliopancreatic diversion [85,86,87].	Metformin, GLP-1 RA (liraglutide, semaglutide, tirzepatide), Contrave, Qsymia, and Orlistat [56,57,58,59,60,61,62,63,64,65,66,67,68,69,70,71,72,73,74,75,76,77,78,79,80,81,82,83,84].
Weight Loss Efficacy	Significant body weight reduction and sustained metabolic improvement.	Variable weight loss [56,57,58,59,60,61,62,63,64,65,66,67,68,69,70,71,72,73,74,75,76,77,78,79,80,81,82,83,84].
Time to Effect	Rapid, with hormonal improvements in 12 months [88].	Depending on medication, GLP-1 RA has a progressive effect over weeks to months [56,57,58,59,60,61,62,63,64,65,66,67,68,69,70,71,72,73,74,75,76,77,78,79,80,81,82,83,84,85].
Safety Considerations	Requires lifestyle changes, surgical risks, and nutritional deficiencies [88].	Risk of specific side effects (teratogenicity with Qsymia, gastrointestinal effects with GLP-1 RA and Orlistat) [56,57,58,59,60,61,62,63,64,65,66,67,68,69,70,71,72,73,74,75,76,77,78,79,80,81,82,83,84].
Preconception Recommendations	Avoid pregnancy for the first 12–24 months post-surgery [88].	Discontinue GLP-1 RA two months before conception; Orlistat has no clear fertility benefits [65,66].

## 10. Current Insights and Special Considerations Around Pharmacological and Surgical Interventions in the Treatment of Infertility in Females with Obesity

Lifestyle modifications represent a first-line and fundamental strategy for improving fertility outcomes in women with obesity. A reduced caloric intake combined with physical activity improves the metabolic state in the context of infertility and obesity. Although pharmacological therapy and bariatric surgery represent effective alternatives, lifestyle changes provide sustainable benefits, at a low cost, without long-term risk, and without teratogenic effects, making them essential for preconceptional evaluation.

In recent years, pharmacological treatment for obesity has proved to be an effective non-invasive option capable of improving fertility outcomes in women with obesity. Moreover, GLP1-RAs have been shown to reduce BMI and IR, exerting favorable effects over ovulatory function and endometrial receptivity. Furthermore, physicians should consider discontinuing treatment prior to conception as phentermine/topiramate has potential teratogenic effects. On the other hand, treatment with Orlistat presents limited efficacy in weight management and infertility. Promising new targeted agent therapies such as twincretins and GLP1-RAs for the management of obesity could possibly impact infertility. However, additional research is required to document their direct benefits on fertility.

Although a wide variety of pharmacological therapies exist for treating obesity, physicians face the challenge of finding a therapeutic option that matches or surpasses the results obtained from bariatric surgery. GLP1-RAs/GIP as a dual therapy has become a novel and key drug for the treatment of obesity and other components of metabolic syndrome. However, no conclusive relationship has been found regarding their impact on female fertility and the process of conception. For this reason, we recommend performing studies on this new therapies in order to elucidate its effects on women who seek to become pregnant or who have infertility problems, taking into account the ethics and well-being of the maternal–fetal binomial.

Obesity can significantly affect fertility outcomes in women of reproductive age, leading to an increased time to conceive, perinatal complications, congenital anomalies, premature births, and fetal death. Thus, effective interventions to treat and prevent infertility in patients with obesity are fundamental. Throughout this review, we have explored the metabolic and reproductive effects of surgical and pharmacological interventions for preconceptional weight management, addressing their benefits and limitations and comparing both types of interventions.

Bariatric surgery presents favorable fertility outcomes by restoring ovulatory function, regulating menstrual cycles, and normalizing hormonal levels. Patients with severe obesity and previous attempts to lose weight with pharmacological interventions and/or lifestyle modifications had a better response with bariatric surgery. Some of the main pathophysiologic mechanisms through which bariatric procedures improve metabolic health include decreased IR and systemic inflammation, both being associated with reproductive dysfunction. Despite the proven benefits, the invasiveness inherent to the procedure and potential complications, such as malnutrition, require careful patient selection and appropriate long-term follow-up.

## 11. Conclusions

The treatment of infertility in women with obesity prompts a multidisciplinary team of endocrinologists, gynecologists, and bariatric specialists to establish tailored treatments to maximize both metabolic health and reproductive outcomes. Further research should be conducted to improve treatment recommendations, elucidate the effect on fertility of combined approaches for weight loss, evaluate long-term effects in maternal and neonatal health, and compare the efficiency of those interventions.

## Data Availability

Not applicable.
